# Acupoints catgut embedding recovers leptin resistance via improving autophagy progress mediated by AMPK-mTOR signaling in obese mice

**DOI:** 10.1016/j.heliyon.2024.e29094

**Published:** 2024-04-06

**Authors:** Youlong Xiong, Xiaoting Wang, Meirong Gong, Qingjie Ji, Yaling Li, Anli Hu, Mengjiang Lu, Bin Xu

**Affiliations:** a Key Laboratory of Acupuncture and Medicine Research of Ministry of Education, Nanjing University of Chinese Medicine, Nanjing 210023, Jiangsu Province, China; b Department of Acupuncture and Moxibustion, Yunnan Provincial Hospital of Traditional Chinese Medicine, Kunming, China; c The Second Clinical Medical College, Yunnan University of Chinese Medicine, Kunming, China; d Department of Rehabilitation, Affiliated Hospital of Jining Medical University, Jining, Shandong Province, China; e Department of Chinese Medicine, Kunming Angel Women's & Children's Hospital, Kunming, China; f Department of Acupuncture and Moxibustion, The Second Affiliated Hospital of Yunnan University of Chinese Medicine, Kunming, China

**Keywords:** Acupoints catgut embedding, Obesity, Leptin resistance, Autophagy, AMPK-mTOR

## Abstract

**Purpose:**

Leptin resistance represents a primary pathological manifestation in obesity. Investigating potential treatments and associated mechanisms to restore leptin sensitivity is crucial for effective obesity management. This study aimed to explore the therapeutic potential of acupoints catgut embedding (ACE) in addressing obesity and its associated leptin resistance.

**Methods:**

A simple obesity model was established by subjecting C57 male mice to a high-fat diet (HFD) for 12 weeks, followed by ACE treatment administered to half of the obese mice for a duration of 4 weeks. The levels of leptin and its receptor-lepRb, were assessed using enzyme-linked immunosorbent assay (ELISA) and Western blot analysis, respectively. Autophagy progression markers were evaluated through quantitative polymerase chain reaction (qPCR) and Western blot analysis. Also, the liver autophagosomes were photographed using electron microscopy. The role of autophagy in regulating leptin resistance was elucidated using an autophagy suppression model.

**Results:**

Comparative analyses demonstrated that ACE treatment resulted in a significant reduction in body weight and blood lipid levels compared to the HFD group. Furthermore, serum leptin levels decreased, while liver lepRb expression increased following ACE treatment. The mRNA and protein expression levels of autophagy in liver were adjusted by ACE treatment. Interestingly, the beneficial effects of ACE were attenuated upon the administration of an autophagy inhibitor. Additionally, ACE treatment led to the activation of the AMPK-mTOR signaling pathway, a crucial regulator of autophagy.

**Conclusion:**

These findings suggest that ACE therapy holds promise for recovering leptin resistance by enhancing autophagy progression, mediated via the AMPK-mTOR signaling pathway in liver.

## 1 Introduction

Obesity has emerged as a global epidemic, with recent statistics revealing a relentless rise in overweight and obesity rates, affecting over two billion individuals, which accounts for approximately 30% of the global population [1]. Leptin, a hormone predominantly secreted by adipose tissue, plays a crucial role in appetite suppression and the regulation of energy balance. Additionally, leptin exhibits pro-inflammatory and platelet aggregation functions. The positive correlation observed between serum leptin levels and body mass index (BMI) highlights the prevalence of leptin resistance in obese individuals, representing a significant pathogenic mechanism in obesity [2][3].

Disruptions in lipid metabolism within the liver play a pivotal role in the development of obesity, and leptin has been implicated in liver function. Experimental models such as the ob/ob and db/db mice, characterized by mutations in the leptin or leptin receptor genes, respectively, exhibit notable alterations in liver function, including impaired lipid tolerance, dyslipidemia, and hepatic steatosis [4]. While recent studies have emphasized the importance of leptin-mediated signaling for maintaining adequate liver function, it remains unclear whether leptin effects are exerted through direct or indirect mechanisms. Notably, liver cells engage in autophagy, a process involved in the degradation of lipid droplets to produce free fatty acids and adenosine triphosphate (ATP), thereby serving as an energy source for the body. Prolonged exposure to a high-fat diet (HFD) promotes lipid accumulation in the liver and suppresses autophagy [5][6].

Acupoints catgut embedding (ACE) is a technique employed in Traditional Chinese Medicine (TCM) that falls under the umbrella of acupuncture. Clinical studies have demonstrated the efficacy of ACE in addressing obesity, with significant reductions in body weight observed after eight sessions of ACE treatment. Furthermore, ACE has shown promise in improving parameters such as triglyceride levels and subcutaneous adipose tissue (SAT) [7][8]. However, the precise mechanism by which ACE induces weight reduction remains unclear.

In the present study, we aimed to investigate the impact of ACE on blood lipid levels and leptin resistance. Moreover, we sought to elucidate the mechanism by which ACE restores leptin receptor sensitivity through the activation of autophagy in the liver. By examining these mechanisms, our study aims to contribute to the understanding of ACE as a potential therapeutic intervention for obesity and its associated metabolic dysregulation.

## 2 Method

### 2.1 Experimental animals and process details

Specific pathogen-free (SPF) C57BL/6 male mice, weighing 10–20g, were procured from Shanghai Slack Animal Co., Ltd. [NO. SCXK (Shanghai) 2017-0002]. The animals were housed at the Experimental Animal Center of Nanjing University of Chinese Medicine. Throughout the study, the mice were maintained under standard laboratory conditions, including an indoor temperature of 22 °C, relative humidity ranging from 40% to 60%, and a 12-h light/dark cycle with alternating illumination. The mice had ad libitum access to food and water.

**Fig. 1:**
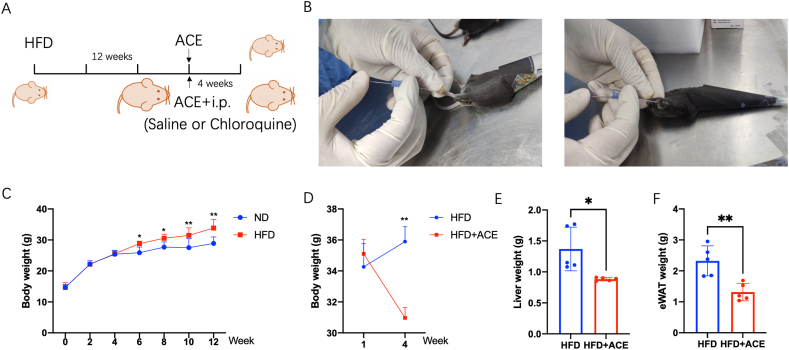
ACE reduce body weight. (A) Schedule of experimental procedures. (B)The surgical operation of ACE treatment in acupoint ST 44 (left) and ST 36(right). (C) Body weight of ND and HFD mice after feeding for 12 weeks(n = 6, *p < 0.05, **p < 0.01). (D) Body weight of HFD mice with or without ACE during 4 weeks after feeding for 12 weeks (n = 6, *p < 0.05). (E) Liver weight in HFD group and HFD + ACE group (n = 5, *p < 0.05). (F) Epididymal white adipose tissue (eWAT) weight in HFD group and HFD + ACE group (n = 5, **p < 0.01).

To establish the obesity model, the mice were fed a high-fat diet (D12492, including Casein, Lactic, Cystine, Lodex 10, Sucrose, Solka Floc, Lard, Soybean Oil and Choline Bitartrate) for a duration of 12 weeks. The criterion for successful model establishment was defined as a body weight increase exceeding 20% compared to the normal diet group, thereby confirming the presence of simple obesity. Thus, the C57BL/6 mice were divided randomly into normal diet and HFD groups. After 12 weeks, the HFD-fed mice were were assigned to several experimental groups: the control group (HFD), the acupoints catgut embedding group (HFD + ACE), the acupoints catgut embedding with additional intraperitoneal injection of saline solvent control group (ACE + Sc), and the acupoints catgut embedding with additional intraperitoneal injection of the autophagy inhibitor Chloroquine(10 mg/kg) group (ACE + Cq). All groups were maintained on a high-fat diet throughout the study. The intraperitoneal injection was once a week and last for 4 weeks (Fig. 1A). At the conclusion of the treatment period, all mice were sacrificed to facilitate the collection of adipose and liver tissues for subsequent analysis.

The mice were euthanized by overdose isoflurane. All experimental procedures were conducted in accordance with the Principles of Laboratory Animal Care and the Guide for the Care and Use of Laboratory Animals, as published by the National Science Council, China (Animal Committee Number: 201902A003).

### 2.2 Acupoints catgut embedding

The animals were subjected to acupoints catgut embedding under anesthesia with isoflurane (0.5%–1.5%). An absorbable surgical suture (Collagen Suture) thread (Jiangxi Longteng Biological High-Tech Co.,Ltd) was cut to a length of 0.3 cm. Subsequently, the thread was inserted into a 6# embedding needle, and the needle was carefully punctured into the acupoints ST36 (Zusanli) and ST44 (Neiting) of the obese mice. The insertion depth of the thread in ST44 is 3 mm, and ST36 is 5 mm. Following insertion, the embedding needle was gently removed, and the thread was secured in place using adhesive tape. The acupoints selected for embedding were ST36, located posterolaterally in the knee, approximately 5 mm below the fibular head, and ST44, situated posterior to the 1st and 2nd metatarsophalangeal joints of both hindlimbs along the Yangming Stomach Meridian (Fig. 1B). The acupoints catgut embedding procedure was performed once a week, for a total of 4 weeks, in accordance with established protocols.

### 2.3 Measurement of serum blood lipid

Serum blood lipid levels were determined using commercially available testing kits (Nanjing Jiancheng, China). For the quantification of free fatty acid, cholesterol, and triglyceride levels, standards and 2.5 μl of mice serum were added to separate wells of a 96-well coated plate, followed by the addition of 250 ml of the working fluid. The plate was then incubated at 37 °C for 10 min. Subsequently, the optical density (OD) values were measured at 510 nm using a microplate reader (Bio-Tek, USA). Quantification of free fatty acid, cholesterol, and triglyceride levels was achieved by constructing standard curves using appropriate reference standards.

### 2.4 RT-PCR

Total RNA was extracted from 100 mg of liver tissue using Trizol (TaKaRa, Japan) following the manufacturer's instructions. The isolated total RNA was then reverse-transcribed into complementary DNA (cDNA) using a thermal cycler (Bio-Rad, United States) and PrimeScript RT Master Mix (TaKaRa, Japan). The reverse transcription reaction was carried out at 42 °C for 15 min, followed by heat inactivation at 85 °C for 15 s. For PCR amplification, a reaction mixture of 20 μL was prepared, comprising 7.2 μL of enzyme-free water, 0.4 μL of a forward primer, 0.4 μL of a reverse primer, 2 μL of the synthesized cDNA, and 10 μL of SYBR Green Mix (TaKaRa, Japan). The PCR protocol involved 40 amplification cycles consisting of denaturation at 95 °C for 30 s, annealing at 95 °C for 5 s, and extension at 60 °C for 30 s. Data analysis was performed using the 2-ΔΔCT method, and the primers utilized in this experiment are provided in Table 1.

**Table 1:** The primary sequence.

Genes	Forward(5′-3′)	Reverse(5′-3′)
*GAPDH*	GAAGGGTGGAGCCAAAAGGG	GACTTCCCGCTCAGATTTCC
*Atg5*	AGAGGAGCCAGGTGATGAT	TTGACTGAAGCAAGGGTGT
*Atg7*	GGGAGAAGAACCAGAAAGGAGG	GCAGGCACTTGACAGACACGAC
*ulk1*	TGCCTCGGTCCCCATTCC	CTGCCAACCCGCACATCA

### 2.5 Western-blot

Total protein was extracted from 100 mg of liver tissue using RIPA lysis buffer (ThermoFisher, USA). The concentration of the extracted protein was determined using the BCA detection method. Subsequently, 50 μg of protein from each sample was separated by sodium dodecyl sulfate-polyacrylamide gel electrophoresis (SDS-PAGE) and transferred onto a nitrocellulose membrane. The membrane was then incubated overnight at 4 °C with primary antibodies targeting Ob-R (1:1000 dilution, Abcam), LC3 (1:1000 dilution, Abcam), p62 (1:1000 dilution, Abcam), mTOR (1:1000 dilution, Abcam), AMPK (1:1000 dilution, Abcam), pAMPK (1:1000 dilution, Abcam), GAPDH (1:1000 dilution, Genetex),and β-actin (1:10000 dilution, Proteintech). Following primary antibody incubation, the membrane was washed and incubated with secondary antibodies, namely goat anti-rabbit IgG-HRP (H + L) and goat anti-mouse IgG-HRP (H + L) (1:10,000 dilution, Signalway Antibody), at room temperature (18–25 °C) for 1 h. Protein bands were visualized using an enhanced chemiluminescence (ECL) detection system.

### 2.6 Electron microscope image

Small liver tissue samples, approximately 2–3 mm in size, were carefully collected and placed in 5 ml of 2.5% glutaraldehyde solution at 4 °C overnight for fixation. Following fixation, the liver samples were subjected to osmium acid fixation, gradient dehydration, infiltration, embedding, trimming, sectioning, and staining processes. Subsequently, the prepared tissue sections were examined using a transmission electron microscope to obtain high-resolution images.

## 3 ELISA

Mouse serum was collected and diluted 20-fold. Subsequently, 50 μL of standard, control, and sample were added to each well of a microplate. The plate was covered with a provided adhesive strip and incubated for 2 h at room temperature on a horizontal orbital microplate shaker set at 500 rpm. Following the incubation period, the contents of each well were aspirated, and a washing step was performed by repeating the process three times, resulting in a total of four washes. Next, 100 μL of Mouse Leptin Conjugate (RD, USA) was added to each well, and the plate was incubated for 1 h at room temperature. To halt the enzymatic reaction, 100 μL of stop solution was added, and the absorbance of the contents in the wells was measured at a wavelength of 450 nm.

## 4 Experimental design and process

### 4.1 Statistical analysis

Statistical analysis was performed using Prism 9.0 software. All data are presented as mean ± standard error (SE) of the mean. For comparisons between two groups, the Student's t-test was utilized. When comparing three groups, one-way analysis of variance (ANOVA) was employed, followed by post hoc Student-Newman-Keuls tests for pairwise comparisons. Statistical significance was defined as a p-value less than 0.05 (*p* < 0.05).

## 5 Results

### 5.1 Acupoints catgut embedding reduce body weight, fat and blood lipids in obesity

**Fig. 2:**
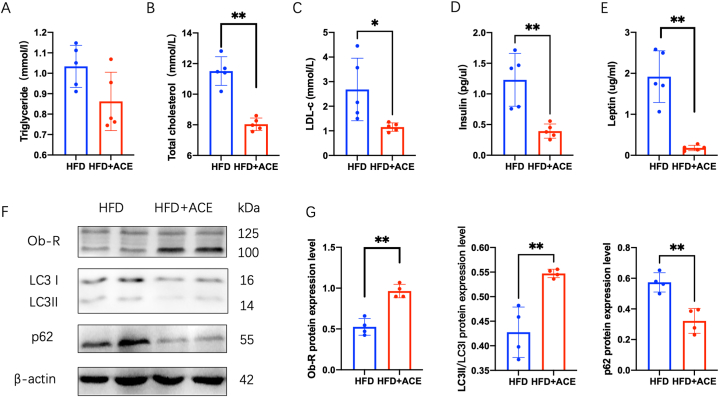
ACE restores blood lipid level and leptin resistance. (A) Serum levels of triglyceride (TG) in the HFD and HFD + ACE groups (*n* = 5).(B) Serum levels of total cholesterol (TC) in the HFD and HFD + ACE groups (n = 5, **p < 0.01). (C) Serum levels of low density lipoprotein cholesterol (LDL-C) in the HFD and HFD + ACE groups (n = 5, *p < 0.05). (D) Serum levels of insulin in the HFD and HFD + ACE groups (n = 5, **p < 0.01). (E) Serum levels of leptin in the HFD and HFD + ACE groups (n = 5, **p < 0.01). (F) Immunoblot image showing Ob-R, LC3 and p62 in the HFD and HFD + ACE groups. (G) Relative Ob-R protein expression in liver (n = 4, **P < 0.01)(left). Relative LC3II/LC3I protein expression in liver (n = 4, **P < 0.01)(middle). Relative p62 protein expression in liver (n = 4, **P < 0.01)(right).

To investigate the therapeutic potential of acupoints catgut embedding (ACE) in obesity, we utilized a well-established obesity model by subjecting mice to a high-fat diet for 12 weeks. Following this, the obese mice received ACE treatment for an additional 4 weeks (Fig. 1A–C). Our results demonstrated a significant reduction in body weight, liver weight and white adipose tissue(WAT) weight following ACE treatment, indicating its efficacy in promoting weight loss in the obese mice(Fig. 1D–F). Furthermore, ACE treatment led to a notable decrease in blood lipid levels, including total cholesterol and low-density lipoprotein cholesterol (LDL-c)(Fig. 2A–C). These findings suggest that ACE intervention has a positive impact on lipid metabolism in obesity. Moreover, ACE treatment exhibited an improvement in insulin and leptin serum level (Fig. 2D and E).

### 5.2 Acupoints catgut embedding regulate leptin resistance and autophagy in liver

**Fig. 3:**
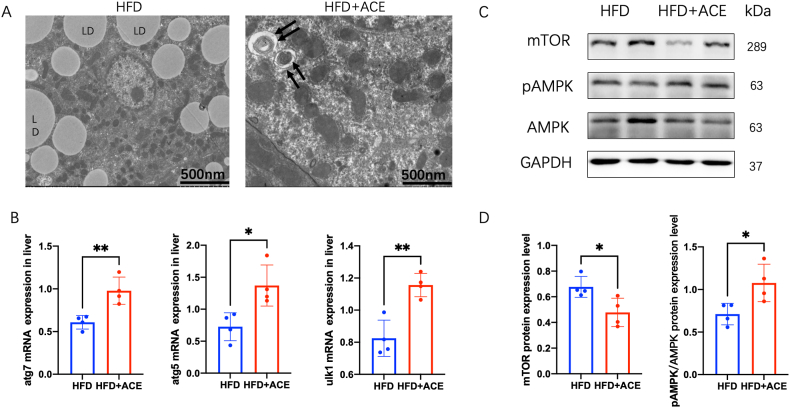
ACE regulates AMPK-mTOR signal to activate autophagy. (A) Liver electron microscope image showing autophagosome and lipid drop (scale bar, 500 nm). The black arrows show autophagosome. (B) Relative atg7 mRNA expression in liver (n = 4, **P < 0.01)(left). Relative atg5 mRNA expression in liver (n = 4, *P < 0.05)(middle). Relative ulk1 mRNA expression in liver (n = 4, **P < 0.01)(right). (C)Immunoblot image showing mTOR, AMPK and *p*-AMPK in the HFD and HFD + ACE groups.(D) Relative mTOR protein expression in liver (n = 4, *P < 0.05)(left). Relative *p*AMPK/AMPK protein expression in liver(n = 4, *P < 0.05)(right).

In obesity, the liver plays a central role in the metabolism of blood lipids and is influenced by leptin resistance [9]. To investigate whether ACE treatment can modulate leptin resistance in the liver, we assessed the expression of the leptin receptor (Ob-R). Notably, ACE treatment significantly upregulated the expression of Ob-R in the liver (Fig. 2F and G). This finding suggests that ACE has the potential to restore leptin receptor sensitivity in the liver, which is essential for the regulation of lipid metabolism. Further investigation has indicated that autophagy is involved in regulating leptin resistance in the liver [10]. We examined the markers of autophagy to determine the impact of ACE treatment on autophagy regulation. As depicted in Fig. 2F and G, ACE treatment led to an increased ratio of LC3II/LC3I, indicating enhanced autophagy activation. Moreover, the ratio of p62, a protein degraded during autophagy, was decreased following ACE treatment(Fig. 2F and G). Consistent with these findings, liver electron microscopy imaging demonstrated the presence of autophagosomes in ACE-treated mice, while HFD mice exhibited lipid droplets (LD) (Fig. 3A). Additionally, ACE treatment resulted in a significant increase in the mRNA expression of autophagy-related genes, including Atg7, Atg5, and Ulk1 in the liver (Fig. 3B). Taken together, our results indicate that acupoints catgut embedding plays a crucial role in the regulation of leptin resistance and autophagy in the liver of obese mice.

### 5.3 Acupoints catgut embedding promote autophagy via AMPK-mTOR signal

**Fig. 4:**
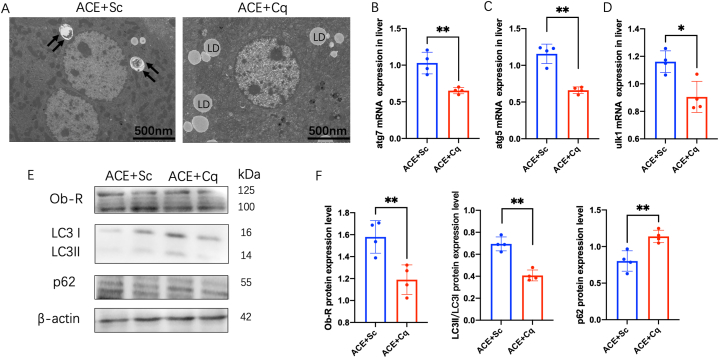
The autophagy inhibitor increases leptin resistance in ACE group.(A) Liver electron microscope image showing autophagosome and lipid drop (scale bar, 500 nm). The black arrows show autophagosome. (B) Relative atg7 mRNA expression in liver (n = 4, **P < 0.01). (C) Relative atg5 mRNA expression in liver (n = 4, **P < 0.01). (D) Relative ulk1 mRNA expression in liver (n = 4, *P < 0.05). (E) Immunoblot image showing Ob-R, LC3 and p62 in the ACE + Sc and ACE + Cq groups. Cq, chloroquine. (F) Relative Ob-R protein expression in liver (n = 4, **P < 0.01)(left). Relative LC3II/LC3I protein expression in liver (n = 4, **P < 0.01)(middle). Relative p62 protein expression in liver (n = 4, **P < 0.01)(right).

Given the pivotal role of AMPK-mTOR signaling in autophagy regulation, we investigated whether ACE treatment influences autophagy through the activation of this signaling pathway. In the liver, ACE treatment led to an increase in AMPK phosphorylation and a decrease in mTOR expression, indicating activation of the AMPK-mTOR signaling cascade (Fig. 3C and D). To further elucidate the affection of autophagy in ACE treatment, we employed Chloroquine (Cq), an autophagy inhibitor, in the obesity mice prior to ACE treatment. Comparative analysis revealed that Cq significantly inhibited the effects of ACE treatment. In the presence of Cq, the liver electron microscopy imaging demonstrated the presence of autophagosomes in ACE-treated mice, while ACE + Cq mice exhibited lipid droplets (LD)(Fig. 4A). The mRNA expression levels of autophagy-related genes, including Atg7, Atg5, and Ulk1, were downregulated in the liver of Cq-treated mice (Fig. 4B–D). Furthermore, the protein expression level of LC3II/LC31 decreased, while p62 expression was upregulated (Fig. 4E and F).

### 5.4 The autophagy in liver controlled leptin resistance and blood lipid

**Fig. 5:**
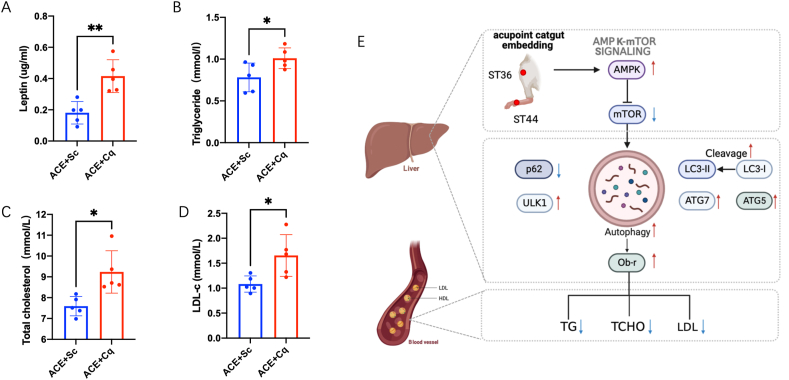
The autophagy inhibitor increases serum lipid level in ACE group. (A)Serum levels of leptin in the ACE + Sc and ACE + Cq groups (n = 5, **p < 0.01). (B)Serum levels of triglyceride (TG) in the ACE + Sc and ACE + Cq groups (n = 5, *p < 0.05). (C) Serum levels of total cholesterol (TC) in the ACE + Sc and ACE + Cq groups (n = 5, *p < 0.05). (D) Serum levels of low density lipoprotein cholesterol (LDL-C) in the ACE + Sc and ACE + Cq groups (n = 5, *p < 0.05). (E) The schematic diagram of the AMPK-mTOR autophagy pathway in lose body weight induced by ACE.

To further investigate the potential involvement of autophagy as an upstream regulator of leptin resistance and lipid metabolism, we examined the expression of the leptin receptor and blood lipid levels in the Cq-treated group. Pre-treatment with Cq, an autophagy inhibitor, was administered prior to ACE treatment. Following Cq administration, a downregulation in the expression level of the leptin receptor (Ob-R) in the liver was observed (Fig. 4E–F). This reduction suggests impaired leptin receptor sensitivity. Furthermore, blood leptin levels were found to be elevated in the Cq-treated mice (Fig. 5A). Moreover, the Cq-treated mice exhibited a significant increase in blood lipid levels, including triglycerides, cholesterol, and low-density lipoprotein cholesterol (LDL-c) (Fig. 5B–D).These findings highlight the potential role of autophagy as an upstream regulator of leptin resistance and lipid metabolism in the liver.

## 6 Discussion

This study aimed to examine the effect of acupoints catgut embedding (ACE) on the mTOR-autophagy-leptin axis in the livers of mice fed a high-fat diet (HFD). Our findings indicate that ACE treatment regulates liver autophagy, resulting in the restoration of leptin receptor sensitivity and improvements in blood lipid levels and weight reduction.

Leptin, a 16 kDa cytokine, was discovered in 1994 through positional cloning. It is primarily synthesized by adipose tissue and released into the bloodstream, circulating in proportion to body fat mass [11]. Leptin signaling is primarily mediated through the leptin receptor (LepRb), which plays a crucial role in transmitting leptin's physiological actions. Decreased sensitivity to leptin is called leptin resistance, and it is commonly observed in obese individuals, involving resistance in both central and peripheral tissues [12]. In peripheral tissues, such as the liver, decreased expression of hepatic LepRb has been observed in obesity, indicative of leptin resistance [13]. However, studies have shown that knockout of hepatic LepRb in mice fed a regular diet does not result in metabolic changes. Conversely, increased expression of hypothalamic LepRb has been speculated to induce hypersensitivity to central leptin action. Notably, liver-specific overexpression of exogenous LepRb in rats deficient in LepRb improves peripheral metabolic abnormalities without affecting food intake [14][15]. These findings suggest that increased endogenous expression of LepRb in the liver could ameliorate peripheral leptin resistance, independent of central leptin resistance. Nonetheless, the regulatory factors governing hepatic LepRb expression and the potential for enhanced peripheral leptin sensitivity to overcome central leptin resistance in obesity remain poorly understood. Acupoints catgut embedding (ACE) is an acupuncture-based therapy rooted in traditional Chinese medicine. This therapeutic approach has garnered significant attention for its potential in addressing obesity. Systematic reviews have systematically assessed the clinical efficacy and safety of ACE in the management of simple obesity [16][17]. Specifically, a 6-week ACE treatment has demonstrated notable reductions in body weight and waist circumference compared to sham catgut embedding interventions. Furthermore, improvements in triglyceride levels and glycohemoglobin have been observed following catgut embedding treatment. The decrease in the leptin-to-adiponectin ratio further signifies an amelioration of leptin resistance in obese patients [18]. In our study, ACE treatment exhibited a decrease in serum leptin levels alongside an increase in hepatic LepRb protein expression, providing evidence for the regulation of endogenous leptin resistance through ACE intervention.

The intracellular storage and utilization of lipids play critical roles in maintaining cellular energy homeostasis. During nutrient deprivation, cellular lipids stored as triglycerides in lipid droplets are hydrolyzed into fatty acids for energy production. Autophagy, induced during starvation, facilitates the degradation of intracellular proteins and organelles by sequestering them within double-membrane vesicles called autophagosomes, which subsequently fuse with lysosomes for degradation and energy utilization. Obesity is characterized by the accumulation of adipose tissue in the liver, leading to the development of a fatty liver condition when fat content exceeds 5% of liver weight [19]. Emerging evidence has implicated autophagy as an essential process for lipid removal in liver cells. The autophagy process involves initiation, maturation/nucleation, and degradation/expansion stages, regulated by various autophagy-related genes (ATGs) [20]. Although at least 41 ATGs have been identified in yeast cells, their homology in mammalian cells is not fully understood[21]. Autophagy is regulated by the formation of the Microtubule-associated protein 1 light chain-3 (LC3) and Beclin-1 complexes [22]. Beclin-1 interacts with the anti-apoptotic Bcl-2 family to modulate autophagy, and the Bcl-2/Beclin-1 complex plays a crucial role in the crosstalk between autophagy and apoptosis [23]. In our study, ACE treatment increased the protein levels of LC3 and Bcl-2 in the livers of mice fed a high-fat diet, suggesting a restoration of autophagy levels. The nutrient-sensing AMPK-mTOR pathway, regulated by PI3K and ULK complexes, serves as a key initiator of autophagy. AMPK directly inhibits mTOR, leading to the suppression of the ULK complex through phosphorylation [24]. Additionally, AMPK directly activates the PI3K complex, initiating autophagosome formation. The elongation step of autophagy requires the presence of ATG5-12-16L and LC3 complexes, which are formed during the ubiquitination process [25]. In our study, ACE treatment activated the AMPK-mTOR signaling pathway, resulting in the inhibition of mTOR expression and an upregulation of downstream autophagy-related mRNA expression (ULK, Atg5, Atg7).Although autophagy and leptin resistance have been associated with obesity, the relationship between autophagy and leptin remains unclear. In our research, we employed chloroquine to inhibit autophagy and observed a decrease in hepatic LepRb expression and a reduction in serum leptin levels. These findings suggest that the suppression of autophagy may contribute to the recovery of leptin resistance in obesity.

## 7 Conclusion

Our research investigated the effects of ACE in the recovery of leptin resistance in obese mice. The mechanism of action of ACE in obesity appears to involve the regulation of autophagy, which may be mediated through the AMPK-mTOR signaling pathway(Fig. 5E). These findings contribute to a deeper understanding of the therapeutic effects of treating obesity.

## Data availability statement

The data are available upon reasonable request to the corresponding author.

## Declaration of conflicts of interest

There are no conflicts of interest to declare.

## Funding

This work was funded by the 10.13039/501100001809National Natural Science Foundation of China (No. 82305375), the Yunnan Provincial Science and Technology Department-Applied Basic Research Joint Special Fund of 10.13039/501100007839Yunnan University of Traditional Chinese Medicine (Grant no. 202001AZ070001-050).

## Ethics approval

The experimental animal research protocol was approved by the Animal Ethics Committee of Nanjing University of Chinese Medicine, ethics approval number: 201902A003.

## CRediT authorship contribution statement

**Youlong Xiong:** Writing – original draft, Funding acquisition. **Xiaoting Wang:** Formal analysis, Data curation. **Meirong Gong:** Methodology, Investigation. **Qingjie Ji:** Software, Resources. **Yaling Li:** Visualization, Validation. **Anli Hu:** Software. **Mengjiang Lu:** Writing – review & editing, Project administration. **Bin Xu:** Resources, Project administration.

## Declaration of competing interest

The authors declare that they have no known competing financial interests or personal relationships that could have appeared to influence the work reported in this paper.
